# Activation tagging in poplar by using an inducible Ac/Ds transposon system

**DOI:** 10.1186/1753-6561-5-S7-O51

**Published:** 2011-09-13

**Authors:** Matthias Fladung, Olaf Polak

**Affiliations:** 1 Johann Heinrich von Thünen Institute (vTI), Institute for Forest Genetics, Sieker Landstr. 2, D-22927 Grosshansdorf, Germany

## Background

The sequence of the whole genome of black cottonwood (*Populus trichocarpa*) was made available in public domain (http://www.phytozome.net/poplar) few years ago. Sequencing and annotation of the genome of an organism yields a tremendous amount of data and information on putative genes, however, often without any idea on their functions. To understand how a cell works one needs to know the function of almost every gene in its genome. Once the whole-genome information is available for an organism, the challenge turns from identifying the parts to understanding their function as well as to improving genome structure. In the short term, the first goal is to assign some element of function to each of the genes in an organism also referred to as ‘functional genomics’, and to do this with high-throughput, systematic approaches.

## Material and methods

Gene technology is a very powerful tool in forest tree breeding programs as well as for functional genomics in order to unraveling gene functions. Various reverse and forward genetics strategies are ongoing to determine the functions of genes and regulatory sequences. A significant progress has been made by using T-DNA as a vehicle to induce either “Knock-out” or “Knock-in” (“gain-of-function” or Activation tagging”) in different plant species including poplar [1].

The application of a transposon-based activation tagging system for poplar has been proposed early by [2]. It could be shown that the maize transposable element *Ac* is functional in the *Populus* genome [3], and re-integrations occur in high frequencies in or near coding regions [4]. Further, the majority of the re-integrations were found scattered over many unlinked sites on other scaffolds than the one carrying the original integration locus, confirming that *Ac* does in fact cross chromosome boundaries in poplar [5].

## Results

In this paper, we describe for the first time the development of an efficient activation tagging system for poplar based on a non-autonomous “Activation Tagging Ds” (ATDs) system [6] in combination with a heat-inducible *Ac*-transposase. First, seven independent transgenic lines were obtained transformed with HSP::*transposase* construct. From these, two transgenic lines were selected for super-transformation with the ATDs-*rolC* construct based on the construct by [6]. The ATDs original integration could be determined from 18 double transgenic lines.

Induction of the transposase gene and mobility of the ATDs element could be confirmed. Four activation-tagged populations comprising in total 12,083 individuals from 22 different ATDs transgenic lines have been produced and phenotyped. So far, from 18 different putatively tagged variants the new ATDs genomic position was successfully determined. Sequences obtained were blasted against the publicly available genome sequence of *P. trichocarpa* v2.0 (Phytozome v5.0; http://www.phytozome.net/poplar). E-values of hits ranged from -35 down to zero. Annotation of the sequences obtained against *P. trichocarpa* revealed for 13 variants possible transcripts. For five putative proteins either no functional annotation or unknown function was found.

In a second approach, 300 randomly selected individuals revealing no obvious phenotype from the forth activation-tagged population were screened for ATDs excision. In approximately one third of the investigated individuals transposition of ATDs was confirmed, and analyses of the new genomic positions of ATDs reveal a very high percentage of tagged genes.

## Conclusions

Tagging approaches based on T-DNA insertion are effective only for plant species (like Arabidopsis and poplar), whose are easily transformable in combination with high frequencies of tagged lines obtained. An advantage of T-DNA based activation tagging could be that even T-DNA insertion sites are not randomly distributed in the genome but do show some insertion site preferences to the 5’UTR of a gene coding region. For transposable elements, however, new insertion sites were found scattered throughout the genome at many unlinked sites. But similar to [5] results for poplar, also other reports describe preferential transposon insertion around transposon donor sites [7].

The fact that a transposon is able to jump to other chromosomes, thus passing chromosomal boundaries, leads to the convenient situation that only a few primary transposon transgenic lines are sufficient for the establishment of large transposon tagging populations in order to tag at least theoretically every gene in a tree genome. This is obviously difficult for T-DNA tagging as plant transformation is time consuming and, therefore, the genome can’t easily be saturated with T-DNA tags.

Taken together, so far the strategy for both T-DNA and transposon activation tagging is first to phenotype an existing tagging population and then to determine the new genomic insertion locus of the tag. A novel strategy of activation tagging can be suggested based on the described power of the ATDs transposon approach and the simplicity to induce ATDs transposition *in vitro*. The ATDs-based strategy allows first the production of a very high number of independent ATDs-transposed plants whose can be screened for new ATDs flanking genomic loci.
